# Automated annular suture device versus conventional annular suture technique in endoscopic aortic valve replacement: A propensity score–matched analysis

**DOI:** 10.1016/j.xjtc.2025.09.034

**Published:** 2025-10-13

**Authors:** Saad Salamate, Ali El-Sayed Ahmad, Ali Bayram, Sami Sirat, Ömür Akhavuz, Mohamed Amer, Jacqueline Kruse, Miriam Silaschi, Mirko Doss, Farhad Bakhtiary

**Affiliations:** a Department of Cardiac Surgery, University Hospital Bonn, Bonn, Germany; b Division of Cardiac Surgery, Heart Centre Siegburg, Siegburg, Germany; c Division of Cardiac Surgery, Helios Klinikum Wuppertal, Wuppertal, Germany

**Keywords:** aortic valve surgery, automated annular suture device, endoscopic aortic valve replacement, endoscopic valve surgery, propensity score matching, right anterior minithoracotomy

## Abstract

**Objective:**

To overcome some of the challenges of endoscopic aortic valve replacement, an automated annular suturing device has been developed and used in aortic valve replacement surgeries. The current study compares the early clinical outcomes of patients who received endoscopic aortic valve replacement with the help of the RAM device (LSI Solutions) versus the conventional annular suture technique.

**Methods:**

From March 2017 to March 2025, 1280 patients underwent endoscopic aortic valve replacement via right anterior minithoracotomy in 3 cardiac referral centers in Germany. The RAM automated suture device was used in 259 cases, and the conventional annular suture technique was used in 1021 patients. A propensity score analysis was performed in 259 matched pairs.

**Results:**

The mean age in the matched cohort was 61.87 ± 11.24 years, and mean body mass index was 27.04 ± 4.83 kg/m^2^ across both genders. Mean aortic crossclamping time was significantly lower in the RAM group: 54.67 ± 16.42 minutes versus 62.02 ± 24.72 minutes (*P <* .001). Mean cardiopulmonary bypass time was also lower in the RAM group (83.75 ± 23.29 minutes vs 97.22 ± 36.45 minutes, *P <* .001). Reexploration for bleeding occurred more often in the conventional suture group (7.3% without RAM vs 2.3% with RAM (*P =* .004). There were no significant differences in the incidence of paravalvular leak between groups (RAM 0% vs conventional 0.4%, *P* = .5).

**Conclusions:**

The use of the RAM device in endoscopic aortic valve replacement is as safe, feasible, and effective as the conventional annular suture technique and yields excellent early outcomes. After a short learning curve, the RAM device was associated with reduced surgical time by facilitating annular suturing in endoscopic fashion.

**Figure:**
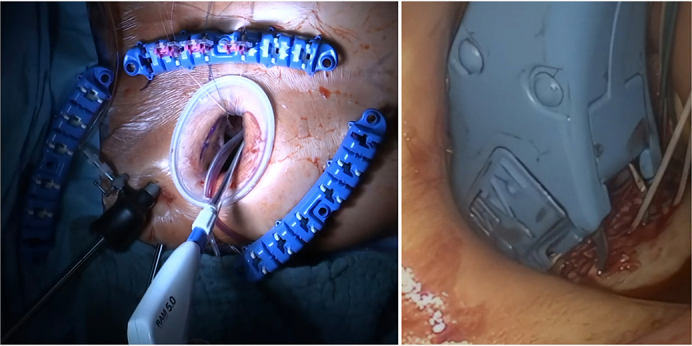
The RAM device being deployed in a totally endoscopic aortic valve surgery.

**Central Message**

The RAM device was associated with shorter operative times, possibly reflecting more efficient annular suturing, without compromising outcomes.

**Perspective**

This propensity-matched analysis demonstrates that the RAM device was associated with faster annular suturing in totally EAVR without compromising outcomes. These findings support broader adoption of automated suturing in minimally invasive valve surgery and may enhance procedural efficiency and reproducibility in clinical practice.

Minimally invasive techniques in valve surgery represent a significant advancement in modern cardiac surgery, offering an effective and safe alternative to traditional median sternotomy. These innovative approaches aim to reduce surgical trauma, improve patient recovery, and enhance clinical outcomes while maintaining the efficacy of conventional procedures.^1^, ^2^, ^3^ Over the past decades, the refinement of surgical instruments, imaging modalities, and video-assisted systems, as well as the development of novel techniques and enhancements in perioperative management, has expanded the applicability of minimally invasive cardiac surgery across a broad spectrum of interventions. These advancements are expected to further improve the safety, precision, and effectiveness of minimally invasive cardiac surgery.^4^, ^5^, ^6^

Although minimally invasive valve surgery offers numerous advantages, its implementation presents distinct challenges. These include the necessity for specialized training due to the sharp learning curve, access to advanced technological resources, and careful patient selection to ensure optimal outcomes. One major obstacle is the endoscopic precise placement of sutures in the valvular annulus within a limited visual and spatial field. To address this issue, the RAM device (LSI Solutions) enhances annular suturing efficiency providing a safe, feasible, and effective solution for the implantation of aortic and mitral valves in fully endoscopic minimally invasive valve surgery through right anterior minithoracotomy (RAMT) with excellent early postoperative outcomes.^7^^,^^8^

This study presents our experience with endoscopic aortic valve replacement (EAVR) surgery via RAMT using an automatic annular suture device and evaluates its early outcomes compared with the endoscopic approach using the conventional annular suturing technique.

## Materials and Methods

### Ethical Approval

The study was approved by the respective Institutional Review Boards (Medical Association of North Rhine Number 82/2021, local ethics board of the University of Bonn Number AZ184/23-EP). Individual patient consent for the study was waived.

### Study Design

The electronic records of all patients who underwent primary isolated aortic valve replacement at 3 cardiac referral centers in Germany (Siegburg Heart Center, Wuppertal Heart Center, and Bonn Heart Center) from March 2017 to March 2025 were retrospectively reviewed for this study. All perioperative data were prospectively collected from the clinic's internal databases, archived patient files, and examination results from referring clinics or practices, and electronically filed and catalogued in the electronic patient record database during the patient's hospital stay; thus, no missing data were encountered. EAVR through RAMT is the first-line treatment strategy for patients selected for isolated aortic valve replacement in the study centers. Before surgery, computed tomography angiography of the aorta and arterial vascular system was performed, and the possibility of a minimally invasive approach was excluded in cases of severe aortic and pelvic artery calcifications, and severe anatomic abnormalities, for example, pronounced severe sternum excavatum or strong adhesions of the lungs. Patients with extensive endocarditis were excluded. The RAM device, designed to facilitate annular suturing in EAVR, uses dual curved needles to place horizontal mattress stitches. It is available in 3.5-mm and 5-mm sizes, chosen based on the valvular ring diameter. Patient selection was determined by surgeon preference and experience with the device, and its use was gradually integrated into clinical practice. The RAM device was used in all 3 participating institutions, with the initial experience at the Siegburg Heart Center and subsequent adoption at the University Hospital Bonn and the Wuppertal Heart Center.

A total of 1280 consecutive patients undergoing EAVR through RAMT were included. Procedures with concomitant coronary artery bypass grafting and ascending aortic surgery, as well as multiple valve surgery, were excluded. Patients were divided into 2 groups: (1) the RAM group, consisting of patients who underwent EAVR using the automated annular suture device; and (2) the conventional group, consisting of patients who underwent EAVR using manually placed pledgeted mattress sutures. Preoperative baseline characteristics of the patients between the 2 groups were then investigated and analyzed.

The primary end point of this study was operation-related mortality, which was defined as in-hospital mortality occurring within 30 postoperative days. Secondary end points were technical success, aortic crossclamping and cardiopulmonary bypass (CPB) times, intensive care unit (ICU) duration and hospital stay, ventilation duration, paravalvular leak, stroke, delirium, acute kidney injury, new-onset persistent atrial fibrillation, and need for pacemaker implantation due to atrioventricular block.

### Operative Techniques

The procedural approach to EAVR via RAMT is based on previously established methodologies and incorporates the RAM device.^4^^,^^9^^,^^10^ The procedure is performed under general anesthesia with endotracheal intubation. Transesophageal echocardiography (TEE) is used in all cases for CPB cannulation guidance and intraoperative cardiac monitoring.

#### Patient preparation and cannulation

The patient is positioned supine with the right hemithorax elevated approximately 20°. After anesthesia induction and single-lumen intubation, a TEE probe is inserted. After sterile preparation, draping, and heparinization, percutaneous femoral artery and vein cannulation is performed using the Seldinger technique under TEE guidance. The MANTA vascular closure device (Teleflex) is used for femoral artery closure postprocedure.^10^

#### Right anterior minithoracotomy access and surgical exposure

A 4-cm incision is made 2 cm lateral to the right sternal border at the third intercostal space. The pleura is entered laterally, preserving the ribs and internal thoracic vessels. A soft tissue retractor enhances exposure. The pericardium is incised 3 cm above the phrenic nerve and extended to the innominate vein cranially and inferior vena cava caudally. Pericardial stay sutures optimize aortic root exposure.

Two 5-mm working ports are placed—1 above the main incision for a 3-dimensional camera (Aesculap Einstein Vision) and 1 laterally for a Chitwood clamp (Scanlan International). Carbon dioxide is insufflated at 3 L/min via the camera port. A Medtronic DLP 9F cardioplegia catheter is inserted in the ascending aorta and secured with a 3-0 polypropylene purse-string suture. It is later used to vent the aortic root. A left ventricular vent is placed via the right upper pulmonary vein.

#### Cardioplegia and aortic valve replacement

The aorta is clamped, and Custodiol cardioplegia is administered antegrade. For significant aortic regurgitation, cardioplegia is delivered into the coronary ostia postarrest. Normothermic CPB is maintained. After aortotomy, 4-0 Prolene stay sutures aid valve exposure. The native valve is excised, and annular calcium is debrided before sizing.

In the RAM group, pledgeted mattress sutures (RAM Cor-Suture Quick Load; LSI Solutions) are placed sequentially using the RAM device. Sutures are loaded into the Sew-Easy Cassette Tip and secured with the Cor-Knot system (LSI Solutions). In the conventional group, pledgeted mattress sutures are placed manually and similarly secured with Cor-Knot.

The aortotomy is closed in 2 layers with 4-0 Prolene. After de-airing, the crossclamp is removed. Temporary pacing wires are inserted, protamine is administered (1:1 with heparin), and valve function is confirmed by TEE.

#### Decannulation and closure

Venous decannulation is done using a circular suture and compression. Arterial closure is achieved with the MANTA device. One pericardial drain and 1 pleural drain are placed via the crossclamp and camera incisions. The ribs are approximated with 2 FiberWire sutures, and the incision is closed in layers.

### Statistical Analysis

Categorical data are expressed as absolute counts n (%). Continuous variables are represented as mean ± SD or as median [interquartile range] depending on normal distribution. The interquartile range is expressed as an interval. Whether a variable had a normal distribution was established via visual inspection of QQ plot and analysis using the Shapiro–Wilk normality test.

**Figure E1:**
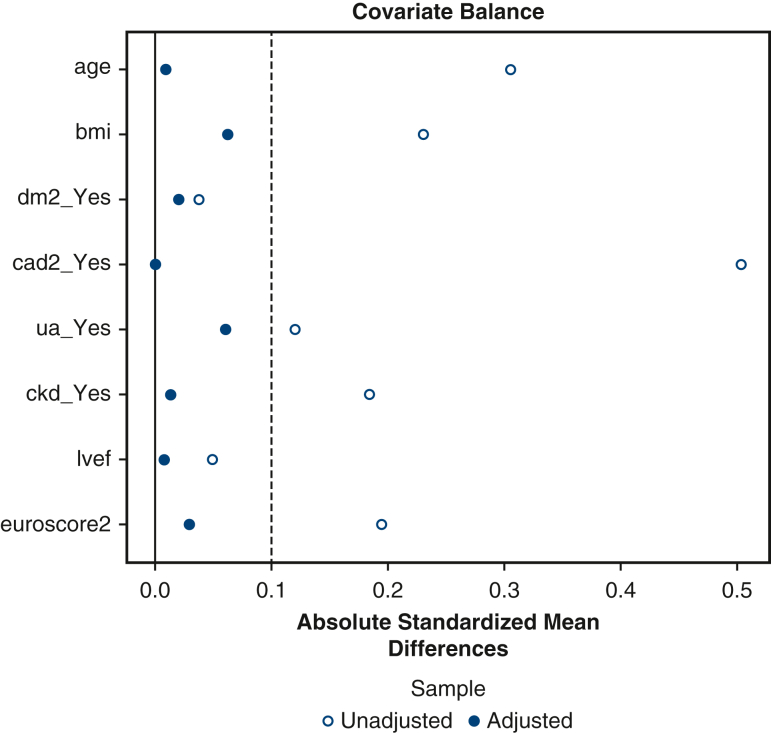
Love plot of absolute SMD for baseline covariates comparing the RAM group with the conventional suturing group before and after propensity score matching.

**Figure E2:**
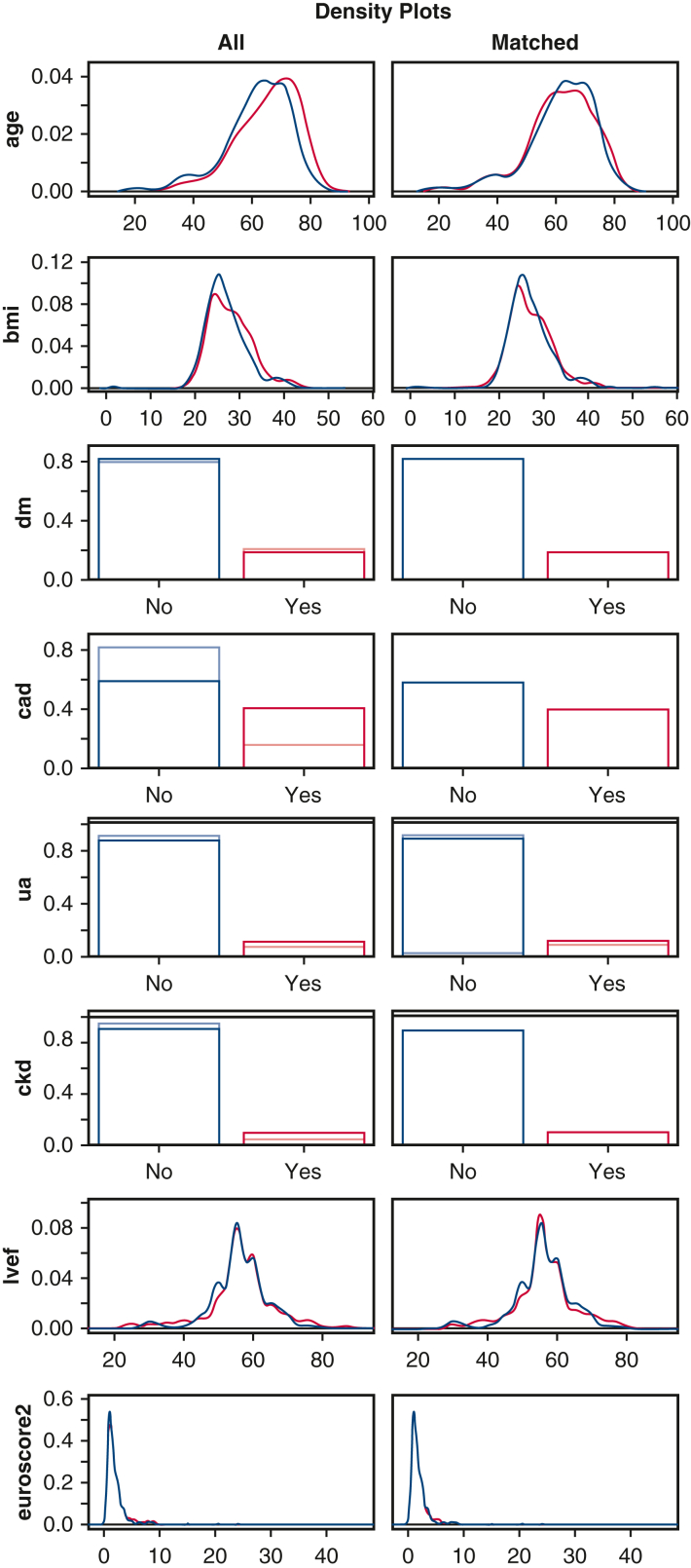
Propensity score density for all matched variables showing distributions of estimated propensity scores by treatment group before and after matching.

Propensity score matching was performed to alleviate the selection bias innate to retrospective studies due to possible confounding factors. The propensity score for each patient was calculated by logistic regression with adjustment for 8 key baseline variables: age, body mass index, diabetes mellitus, coronary artery disease, unstable angina, chronic kidney disease, heart failure, and European System for Cardiac Operative Risk Evaluation II. Propensity score matching was performed on all 259 patients in the RAM group using 1:1 nearest neighbor matching without replacement, resulting in successful matching of all cases. Because of the large control pool, common support was complete, and a caliper was not required. The Love plot and propensity score density are shown in Figures E1 and E2, respectively. After matching, absolute standardized mean differences (SMDs) were used to assess the balance between the 2 groups, with acceptable difference in means being less than 0.1.

Comparisons of continuous outcome variables before matching were done with the nonparametric Mann–Whitney test, and categorical variables were compared using Pearson's test and Fisher exact test where appropriate, depending on whether the minimum expected assumption was met. Comparisons between the 2 matched groups were carried out using a paired *t* test or the Wilcoxon signed rank-sum test for continuous variables, depending on the normality of the distribution. Categorical variables were compared using the McNemar's mid-p test. All statistical analyses was performed using the R statistical software (R Foundation for Statistical Computing). The MatchIt package was used for propensity score matching.

## Results

### Patient Characteristics

A total of 1280 patients underwent minimally invasive aortic valve replacement, with 259 (20.2%) receiving the RAM device and 1021 (79.8%) undergoing conventional annular suturing. Before propensity matching, patients in the RAM group were significantly younger (61.92 ± 11.07 vs 65.31 ± 10.6 years, SMD = 0.312), had lower body mass index (26.9 ± 4.72 vs 27.99 ± 4.94, SMD = 0.226), had higher rates of hypertension (76.4% vs 54.4%, SMD = 0.477), and more frequently presented with bicuspid aortic valves (33.2% vs 5.8%, SMD = 0.738). After propensity matching, baseline characteristics were well balanced between the RAM (n = 259) and conventional suturing groups (n = 259), with an SMD less than 0.1 for most variables except for notable residual differences in hypertension (76.4% vs 57.5%, SMD = 0.411) and bicuspid aortic valves (33.2% vs 9.7%, SMD = 0.599). Patient characteristics before and after propensity matching are summarized in Tables 1 and 2.

**Table 1:** Patient characteristics before matching

Variable	Overall (N = 1280)	No RAM (N = 1021)	RAM (N = 259)	SMD
Age,∗y (mean ± SD)	64.62 ± 10.78	65.31 ± 10.6	61.92 ± 11.07	0.312
BMI∗ (mean ± SD)	27.76 ± 4.91	27.99 ± 4.94	26.9 ± 4.72	0.226
EuroSCORE II∗ (median [IQR])	1.56 [1.56-2.45]	1.57 [1.08-2.52]	1.34 [0.9-2.38]	0.148
LVEF∗ (median [IQR])	55 [54-60]	55 [54-60]	55 [52.5-60]	0.043
Female (%)	424 (33.1%)	359 (35.2%)	65 (25.1%)	0.221
Obese (%)	374 (29.2%)	320 (31.3%)	54 (20.8%)	0.241
CAD∗ (%)				0.582
Single disease	169 (13.2%)	105 (10.3%)	64 (24.7%)	
Double disease	64 (5%)	33 (3.2%)	31 (12)	
Triple disease	38 (3%)	27 (2.6%)	11 (4.2%)	
None	1009 (78.8%)	856 (83.8%)	153 (59.1%)	
Unstable angina∗ (%)	109 (8.5%)	79 (7.7%)	30 (11.6%)	0.130
HTN (%)	753 (58.8%)	555 (54.4%)	198 (76.4%)	0.477
Smoker (%)				0.565
Ex-smoker	17 (1.3%)	16 (1.6%)	1 (0.4%)	
No	1064 (83.1%)	882 (86.4%)	182 (70.3%)	
Yes	199 (15.5%)	123 (12%)	76 (29.3%)	
Diabetes∗ (%)	242 (18.9%)	196 (19.2%)	46 (17.8%)	0.037
CKD∗ (%)	72 (5.6%)	46 (4.5%)	26 (10%)	0.214
COPD (%)	132 (10.3%)	104 (10.2%)	28 (10.8%)	0.020
Carotid stenosis (%)	47 (3.7%)	25 (2.4%)	22 (8.5%)	0.268
PAD (%)	65 (5.1%)	47 (4.6%)	18 (6.9%)	0.101
Elective surgery (%)	1250 (97.7%)	994 (97.4%)	256 (98.8%)	0.109
Pulmonary HTN (%)	41 (3.2%)	33 (3.2%)	8 (3.1%)	0.008
Previous heart surgery (%)	7 (0.5%)	3 (0.3%)	4 (1.5%)	0.131
Aortic stenosis (%)	572 (44.7%)	495 (48.5%)	77 (29.7%)	0.392
Aortic insufficiency (%)	151 (11.8%)	112 (11%)	39 (15.1%)	0.122
Combined aortic vitium	557 (43.5%)	414 (40.5%)	143 (55.2%)	0.297
Bicuspid aortic valve (%)	145 (11.3%)	59 (5.8%)	86 (33.2%)	0.738
Biological prosthesis (%)	1202 (93.9%)	945 (92.6%)	257 (99.2%)	0.341

All values are represented as absolute number N (%) unless specified otherwise. *SMD*, Standardized mean difference; *BMI*, body mass index; *EuroSCORE*, European System for Cardiac Operative Risk Evaluation; *IQR*, interquartile range; *LVEF*, left ventricular ejection fraction; *CAD*, coronary artery disease; *HTN*, hypertension; *CKD*, chronic kidney disease; *COPD*, chronic obstructive pulmonary disease; *PAD*, peripheral artery disease.

∗ Variables used in propensity score matching.

**Table 2:** Patient characteristics after matching

Variable	Overall (N = 518)	No RAM (N = 259)	RAM (N = 259)	SMD
Age,∗ y (mean ± SD)	61.87 ± 11.24	61.82 ± 11.42	61.92 ± 11.07	0.009
BMI∗ (mean ± SD)	27.04 ± 4.83	27.19 ± 4.94	26.90 ± 4.72	0.061
EuroSCORE II∗ (median [IQR])	1.4 [0.9-2.35]	1.44 [0.9-2.26]	1.34 [0.9-2.38]	0.028
LVEF∗ (median [IQR])	55 [54-60]	55 [54-60]	55 [52.5-60]	0.007
Female (%)	140 (27%)	75 (29%)	65 (25.1%)	0.087
Obese (%)	119 (23%)	65 (25.1%)	54 (20.8%)	0.1
CAD∗ (%)				<0.001
Single disease	130 (25.1%)	66 (25.5%)	64 (24.7%)	
Double disease	52 (10%)	21 (8.1%)	31 (12%)	
Triple disease	30 (5.8%)	19 (7.3%)	11 (4.2%)	
None	306 (59.1%)	153 (59.1)	153 (59.1)	
Unstable angina∗ (%)	34 (16%)	15 (14.2%)	19 (17.9%)	0.1
HTN (%)	347 (67%)	149 (57.5%)	198 (76.4%)	0.411
Smoker (%)				0.336
Ex-smoker	4 (0.8%)	3 (1.2%)	1 (0.4%)	
No	397 (76.6%)	215 (83%)	182 (70.3%)	
Yes	117 (22.6%)	41 (15.8%)	76 (29.3%)	
Diabetes∗ (%)	90 (17.4%)	44 (17%)	46 (17.8%)	0.020
CKD∗ (%)	24 (11.3%)	13 (12.3%)	11 (10.4%)	0.060
COPD (%)	56 (10.8%)	28 (10.8%)	28 (10.8%)	<0.001
Carotid stenosis (%)	30 (5.8%)	8 (3.1%)	22 (8.5%)	0.233
PAD (%)	38 (7.3%)	20 (7.7%)	18 (6.9%)	0.030
Elective surgery (%)	509 (98.3%)	253 (97.7%)	256 (98.8%)	0.089
Pulmonary HTN (%)	20 (3.9%)	12 (4.6%)	8 (3.1%)	0.080
Previous heart surgery (%)	5 (1%)	1 (0.4%)	4 (1.5%)	0.119
Aortic stenosis (%)	190 (36.7%)	113 (43.6%)	77 (29.7%)	0.291
Aortic insufficiency (%)	65 (12.5%)	26 (10%)	39 (15.1%)	0.152
Combined aortic vitium	263 (50.8%)	120 (46.3%)	143 (55.2%)	0.178
Bicuspid aortic valve (%)	111 (21.4%)	25 (9.7%)	86 (33.2%)	0.599
Biological prosthesis (%)	482 (93.1%)	225 (86.9%)	257 (99.2%)	0.501

All values are represented as absolute number N (%) unless specified otherwise. *SMD*, Standardized mean difference; *BMI*, body mass index; *EuroSCORE*, European System for Cardiac Operative Risk Evaluation; *IQR*, interquartile range; *LVEF*, left ventricular ejection fraction; *CAD*, coronary artery disease; *HTN*, hypertension; *CKD*, chronic kidney disease; *COPD*, chronic obstructive pulmonary disease; *PAD*, peripheral artery disease.

∗ Variables used in propensity score matching.

### Intraoperative Outcomes

**Figure 1:**
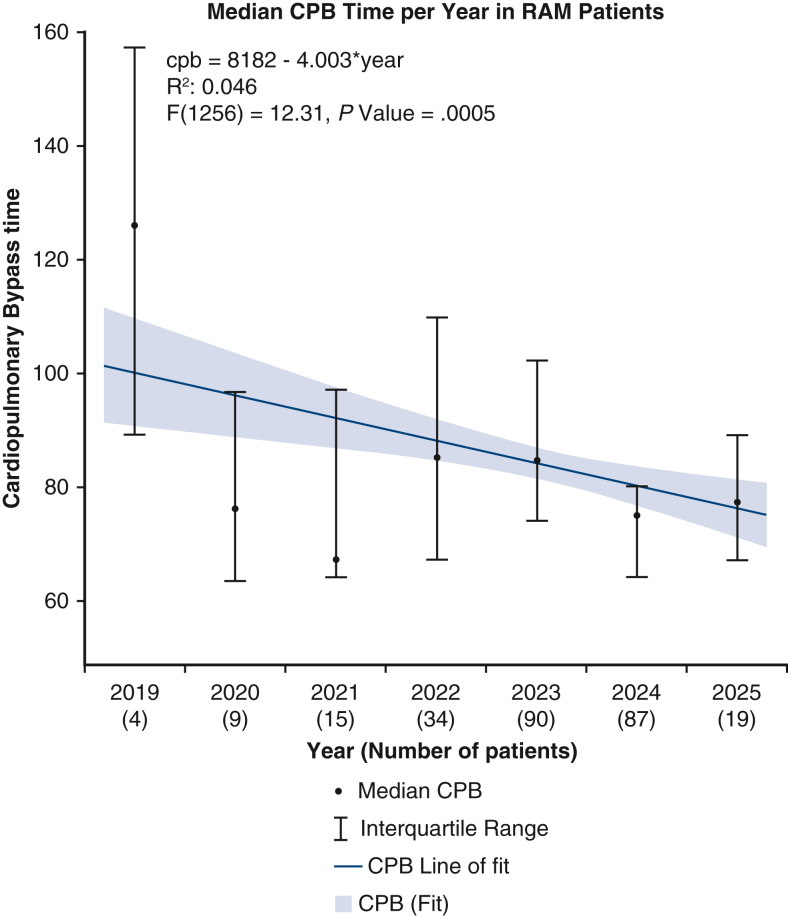
Median CPB time per year in RAM group. Error bars represent interquartile ranges. *Blue line* shows simple univariate linear regression. Patient numbers for each year are shown in parentheses. *CPB*, Cardiopulmonary bypass.

**Figure 2:**
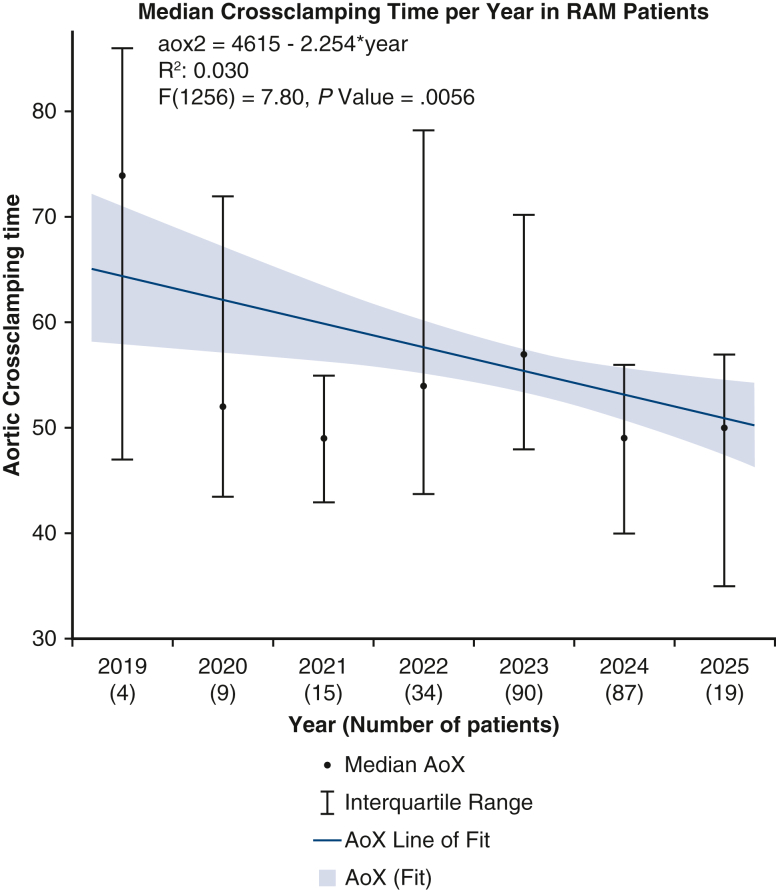
Median aortic crossclamping time per year in RAM group. Error bars represent interquartile ranges. *Blue line* shows simple univariate linear regression. Patient numbers for each year are shown in parentheses. *XoX*, Aortic crossclamp.

**Figure 3:**
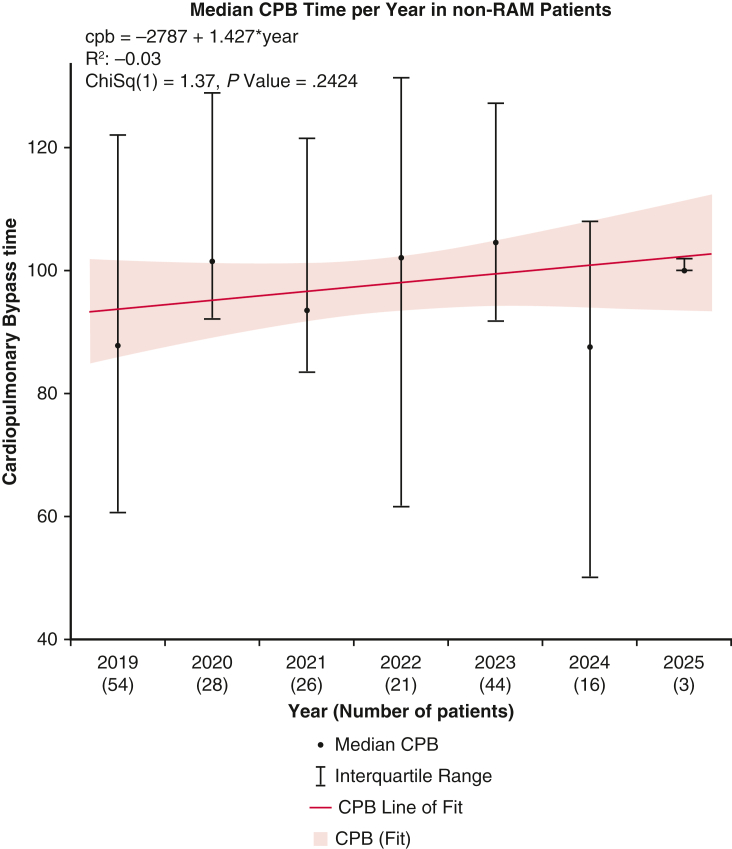
Median CPB time per year in non-RAM group. Error bars represent interquartile ranges. *Red line* shows simple univariate linear regression. Patient numbers for each year are shown in parentheses. *CPB*, Cardiopulmonary bypass.

**Figure 4:**
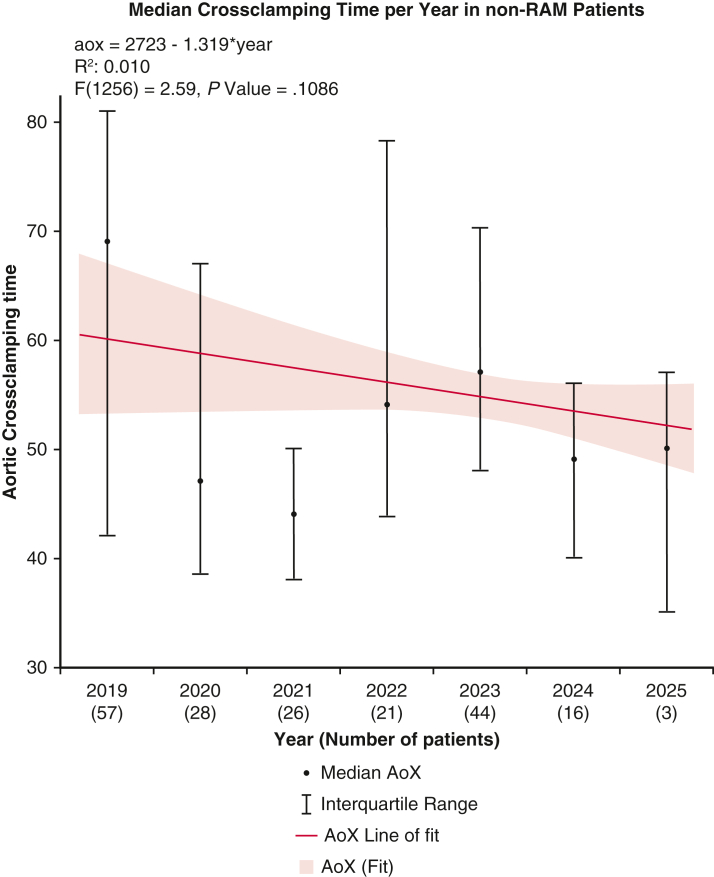
Median aortic crossclamping time per year in non-RAM group. Error bars represent interquartile ranges. *Red line* shows simple univariate linear regression. Patient numbers for each year are shown in parentheses. *XoX*, Aortic crossclamp.

After propensity matching, the RAM group demonstrated significantly shorter CPB times (83.75 ± 23.29 minutes vs 97.22 ± 36.45 minutes, *P <* .001) and reduced aortic crossclamping times (54.67 ± 16.42 minutes vs 62.02 ± 24.72 minutes, *P <* .001) compared with conventional suturing. A simple univariate linear regression analysis demonstrated a significant decrease in median CPB and aortic crossclamping times over the study period (Figures 1 and 2). For comparison, Figures 3 and 4 show no significant temporal change in median CPB and crossclamping times in patients from the conventional sutures group during the same period. Intraoperative outcomes both before and after propensity matching are shown in Table 3.

**Table 3:** Postoperative outcome before and after matching

Variable	Nonmatched groups			Matched groups		
	No RAM (N = 1021)	RAM (N = 259)	*P*	No RAM (N = 259)	RAM (N = 259)	*P*
ICU stay, d (median [IQR])	1 [1-2]	1 [1-2]	.001	1 [1-2]	1 [1-2]	.001
Hospital stay, d (median [IQR])	8 [7-11]	7 [6-8]	<.001	8 [7-11]	7 [6-8]	<.001
CPB time, min (mean ± SD)	94.16 ± 34.4	83.75 ± 23.29	<.001	97.22 ± 36.45	83.75 ± 23.29	<.001
Aortic clamping, min (mean ± SD)	59.43 ± 23.41	54.67 ± 16.42	.002	62.02 ± 24.72	54.67 ± 16.42	<.001
Myocardial infarct (%)	4 (0.4%)	1 (0.4%)	1	3 (1.2%)	1 (0.4%)	.375
Respiratory insufficiency (%)	35 (3.4%)	7 (2.7%)	.558	17 (6.6%)	7 (2.7%)	.043
Acute kidney injury (%)	17 (1.7%)	4 (1.5%)	1	7 (2.7%)	4 (1.5%)	.388
Permanent dialysis (%)	6 (0.6%)	0 (0%)	.607	4 (1.5%)	0 (0%)	.062
Reexploration for thoracic bleeding (%)	33 (3.2%)	6 (2.3%)	.444	19 (7.3%)	6 (2.3%)	.007
Delirium (%)	68 (6.7%)	12 (4.6%)	.229	22 (8.5%)	12 (4.6%)	.09
TIA (%)	5 (0.5%)	0 (0%)	.590	2 (0.8%)	0 (0%)	.25
Stroke (%)	22 (2.2%)	1 (0.4%)	.065	8 (3.1%)	1 (0.4%)	.021
Deep wound healing disorder (%)	7 (0.7%)	1 (0.4%)	1	2 (0.8%)	1 (0.4%)	.625
Wound revision (%)	6 (0.6%)	0 (0%)	.607	1 (0.4%)	0 (0%)	.5
Persistent atrial fibrillation (%)	11 (1.1%)	0 (0%)	.134	1 (0.4%)	0 (0%)	.5
Pacemaker implantation due to AVB (%)	11 (1.1%)	2 (0.8%)	1	2 (0.8%)	2 (0.8%)	1
Pleural effusion (%)	51 (5%)	17 (6.6%)	.315	24 (9.3%)	17 (6.6%)	.243
Pleural effusion requiring invasive treatment (%)	40 (3.9%)	6 (2.3%)	.216	16 (6.2%)	6 (2.3%)	.035
Paravalvular leak (%)	7 (0.7%)	0 (0%)	.551	1 (0.4%)	0 (0%)	.5
30-d mortality (%)	16 (1.6%)	0 (0%)	.054	8 (3.1%)	0 (0%)	.004

All values are represented as absolute number N (%) unless specified otherwise. *ICU*, Intensive care unit; *IQR*, interquartile range; *CPB*, cardiopulmonary bypass; *TIA*, transient ischemic attack; *AVB*, atrioventricular block.

### Postoperative Outcomes

Before matching, patients in the RAM group showed significantly shorter ICU stays (median 1 [1-2] days vs 1 [1-2] days, *P =* .001) and hospital stays (7 [6-8] days vs 8 [7-11] days, *P <* .001). Patients in the RAM group also experienced statistically similar rates of reexplorations for thoracic bleeding (2.3% vs 3.2%, *P =* .444) and strokes (0.4% vs 2.2%, *P =* .065). Pacemaker implantation due to aortic valve block was comparable (RAM: 0.8% vs conventional: 1.1%, *P* = 1). Permanent dialysis was not required in the RAM group versus 0.6% in the conventional group (*P =* .607).

After propensity matching, the RAM group continued to show significantly shorter ICU and hospital stays despite similar median values, explained by differing distributions confirmed by Wilcox signed rank-sum tests. Patients in the RAM group had significantly fewer cases of respiratory insufficiency (2.7% vs 6.6%, *P =* .043), reexplorations for thoracic bleeding (2.3% vs 7.3%, *P =* .007), strokes (0.4% vs 3.1%, *P =* .021), and pleural effusion requiring invasive treatment (2.3% vs 6.2%, *P =* .035). The 30-day mortality was significantly lower in the RAM group (0% vs 3.1%, *P =* .004). There were no significant differences in the incidence of paravalvular leak between groups both intraoperatively and at early postoperative assessment (RAM 0% vs conventional 0.4%, *P =* .5).

## Discussion

The results of this propensity-matched analysis demonstrate clear benefits of using the RAM device in minimally invasive aortic valve replacement, including reduced operative times and improved postoperative outcomes. The significantly shorter CPB and aortic crossclamping times observed in the RAM group are likely associated with procedural efficiency and technical precision associated with automated suturing, potentially contributing to the reduced incidence of postoperative complications. These findings support the integration of RAM into EAVR, particularly as the field advances toward greater efficiency and reproducibility.

### Operative Efficiency and Crossclamp Time

One of the most notable findings in this study is the reduction in CPB and aortic crossclamping times in the RAM group after matching. These time savings are clinically significant. Prolonged aortic crossclamping has long been associated with increased morbidity and mortality in cardiac surgery, even in low-risk cohorts.^11^ By mitigating these risks, the RAM device could indirectly contribute to improved patient recovery.

Our findings echo those from our previous observational studies reporting reduced operative times with the RAM device in totally endoscopic valve surgery.^8^ Given that the annular suturing technique was the only procedural difference between groups, the reduction in crossclamp and CPB times may reflect greater efficiency of the automated device, although annular suturing time was not measured directly, we hypothesize that device-facilitated annular suturing contributed to the observed time saving. The ability to perform secure annular suturing reproducibly may help overcome a persistent barrier to wider adoption of minimally invasive cardiac surgery. Furthermore, linear regression analyses demonstrated a continued decline in operative times over the study period, highlighting both the impact of cumulative experience and the growing procedural efficiency facilitated by the device.

### Clinical Outcomes and Safety

The improved operative efficiency did not come at the cost of patient safety. After matching, patients in the RAM group experienced significantly fewer postoperative complications including respiratory insufficiency, reexploration for thoracic bleeding, stroke, and pleural effusion requiring invasive treatment.

Although median ICU and hospital stays were similar, Wilcoxon signed-rank tests confirmed significantly shorter durations for patients in the RAM group, suggesting not only faster recovery in some patients but also a more consistent postoperative course.

The lower 30-day mortality observed in the RAM group further supports its safety and effectiveness. Notably, there were no differences in the rates of pacemaker implantation due to aortic valve block, suggesting that the RAM device does not increase the risk of conduction system injury. Although differences in acute kidney injury and permanent dialysis rates did not reach statistical significance, both outcomes were lower in the RAM group. This aligns with prior literature linking prolonged ischemic and crossclamp times to increased renal injury, further reinforcing the benefit of shorter operative times observed with the RAM device.^11^^,^^12^ Likewise, prolonged crossclamp and CPB times have been associated with an increased risk of postoperative bleeding and reexploration in cardiac surgery. In our series, all bleeding reexplorations were performed through the initial endoscopic access without conversion to sternotomy, and the shorter operative times observed with the RAM device may also contribute to the lower rate of reexplorations for bleeding.^11^^,^^13^^,^^14^ Although residual confounding cannot be fully excluded despite propensity score matching, these time-related effects likely explain part of the observed difference.

### Integration Into the Evolution of Minimally Invasive Cardiac Surgery

The advancement of devices such as the RAM must be viewed within the broader context of a field steadily moving toward micro-invasive approaches. As described in our state-of-the-art micro-invasive cardiac surgery, endoscopic cardiac surgery now includes complex procedures such as double valve replacements, root surgery, and reoperations, all facilitated by precision tools and 3-dimensional visualization.^5^ In this setting, reducing procedural variability through standardized suturing could support broader adoption of advanced techniques, reduce training curves, and minimize human error.

### Limitations

Although our study adds to the growing evidence base on the RAM device, it is limited by its retrospective design. Despite propensity scoring, residual confounding may exist. Propensity score matching did not account for surgeon experience, institutional differences, or detailed aortic valve pathoanatomy (annular calcification severity, annular size), which were not consistently quantified across centers. Moreover, residual imbalances persisted for some baseline features (eg, hypertension, bicuspid valve), and unmeasured factors influencing surgeon choice may remain. Additionally, long-term durability of annular sutures placed with automated devices will require longitudinal follow-up.

Because annular suturing time was not recorded, we cannot ascribe causality. We hypothesize that shorter crossclamp and CPB times with the RAM device may reflect greater efficiency of annular suturing; however, alternative explanations—including institutional learning, workflow changes, and center-level practices—may contribute. Thus, the observed differences should be cautiously interpreted.

Cost data were not collected as part of this retrospective analysis; therefore, a cost-effectiveness comparison between the RAM device and conventional suturing could not be performed. Future prospective studies are needed to address this important aspect.

Future studies should explore the learning curves, cost-effectiveness, and impact of RAM use on resource use such as operative turnover, ICU stay, and hospital readmission. In addition, integration into more complex procedures such as root replacement, as explored by Hamiko and colleagues,^15^ could further extend the application spectrum of automated suturing in endoscopic surgery.

## Conclusions

The RAM automated suturing device offers a safe and efficient alternative to conventional annular suturing in totally EAVR and was associated with reduced operative times without compromising short-term clinical outcomes. As minimally invasive approaches continue to evolve, technologies that simplify complex steps without sacrificing safety will be instrumental in broadening the accessibility and consistency of advanced cardiac surgery.

### Webcast

You can watch a Webcast of this AATS meeting presentation by going to: https://www.aats.org/resources/comparison-of-automated-annula-10040.

**Figure:**
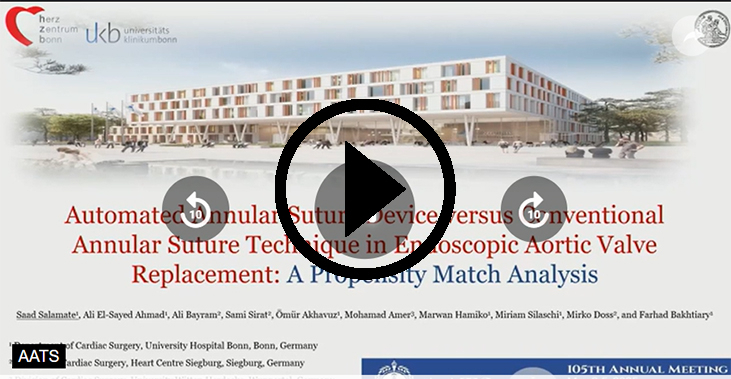

### Audio

You can listen to the discussion audio of this article by going to the supplementary material section below.

## Conflict of Interest Statement

F.B. reports a relationship with Edwards Lifesciences, Medtronic, Corcym, and Abbott that includes consulting or advisory and speaking and lecture fees; and a relationship with LSI that includes speaking and lecture fees. All other authors reported no conflicts of interest.

The *Journal* policy requires editors and reviewers to disclose conflicts of interest and to decline handling or reviewing manuscripts for which they may have a conflict of interest. The editors and reviewers of this article have no conflicts of interest.

## Supplementary Data

### Audio

Audio 1 This is an audio recording of the presentation of the associated Abstract and Discussion. The Abstract presentation ends at 10:15, and the Discussion begins at 10:18.2

Video 1 Video available at: https://www.jtcvs.org/article/S2666-2507(25)00457-2/fulltext.1
